# Merged Lateral Row Modification of the Suture Bridge Technique for Simultaneous Supraspinatus and Subscapularis Repair

**DOI:** 10.1016/j.eats.2022.08.054

**Published:** 2022-12-21

**Authors:** Yu-Cheng Su, Yu-Ju Lin, Wen-Hao Chang, Jou-Hua Wang, Kai-Lan Hsu, Fa-Chuan Kuan, Wei-Ren Su

**Affiliations:** a Department of Orthopaedic Surgery, National Cheng Kung University Hospital, College of Medicine, National Cheng Kung University, Tainan, Taiwan; b Department of Biomedical Engineering, National Cheng Kung University, Tainan, Taiwan; c Division of Traumatology, National Cheng Kung University Medical Center, Tainan, Taiwan; d Skeleton Materials and Bio-compatibility Core Lab, Research Center of Clinical Medicine, National Cheng Kung University Hospital, College of Medicine, National Cheng Kung University, Tainan, Taiwan; e Division of Orthopaedics, Department of Surgery, National Cheng Kung University Hospital Dou Liou Branch, National Cheng Kung University, Yunlin, Taiwan

## Abstract

Concomitate supraspinatus and subscapularis tear is not rare, and the suture bridge technique is one of the most effective methods for rotator cuff repair. However, some limitations exist in the use of such a technique for simultaneous supraspinatus and subscapularis repair. We introduce the technique of a merged lateral row for suture bridge rotator cuff repair, in which the lateral suture of the supraspinatus and subscapularis is placed in the greater tuberosity. We believe that this technique can reduce both the duration and cost of surgery and decrease soft-tissue damage. It can also allow the “comma tissue,” to be simultaneously repaired.

With advances in the diagnosis of subscapularis tear, simultaneous supraspinatus and subscapularis tear have become more common.^1^ Although the suture bridge technique, also called transosseous-equivalent repair, is one of the most effective methods for rotator cuff repair,^2^ some limitations exist in the use of such a technique for simultaneous supraspinatus and subscapularis repair.

First, to use the suture bridge technique in cases of subscapularis tear, the knotless suture anchor in the lateral row should be placed in the most superior side of the bicipital groove.^2^ Thus, parts of the bicipital pulley, including the coracohumeral ligament and superior glenohumeral ligament (SGHL), should be debrided and removed. Second, to use the suture bridge technique for both the supraspinatus and subscapularis tendons, 4 knotless suture anchors in the lateral row are necessary,^3^ which may increase the duration and cost of surgery and the risk of future fractures.

Therefore, we introduce the technique of a merged lateral row for suture bridge rotator cuff repair, in which the lateral suture of the supraspinatus and subscapularis is placed in the greater tuberosity. We believe that this technique can reduce both the duration and cost of surgery and decrease soft-tissue damage. It also can allow the displaced coracohumeral ligament and SGHL, known as the “comma tissue,” to be simultaneously repaired.

## Surgical Technique (With Video Illustration)

The report was approved by National Cheng Kung University Hospital institutional review board (B-ER-109-428). A detailed video with a demonstration of the surgical technique described in this article may be reviewed (Video 1).

### Patient Positioning, Portal Placement, and Diagnostic Arthroscopy

Surgery is performed with the patient under general anesthesia with an additional interscalene block. The patient is placed in the lateral decubitus position with index arm traction by a 3-point traction system (Arthrex, Naples, FL). Subsequently, after aseptic preparation and draping, a standard posterior viewing portal is established. Thereafter, a standard anterior portal is created using spinal needle localization immediately lateral to the tip of the coracoid, and a probe is introduced. Glenohumeral diagnostic arthroscopy is then performed using a standard posterior portal with a 30° scope. Generally, tenotomy of the long head of the biceps is usually performed unless in a young patient.

### Visualization, Mobilization, and Repair of the Subscapularis

**Fig 1:**
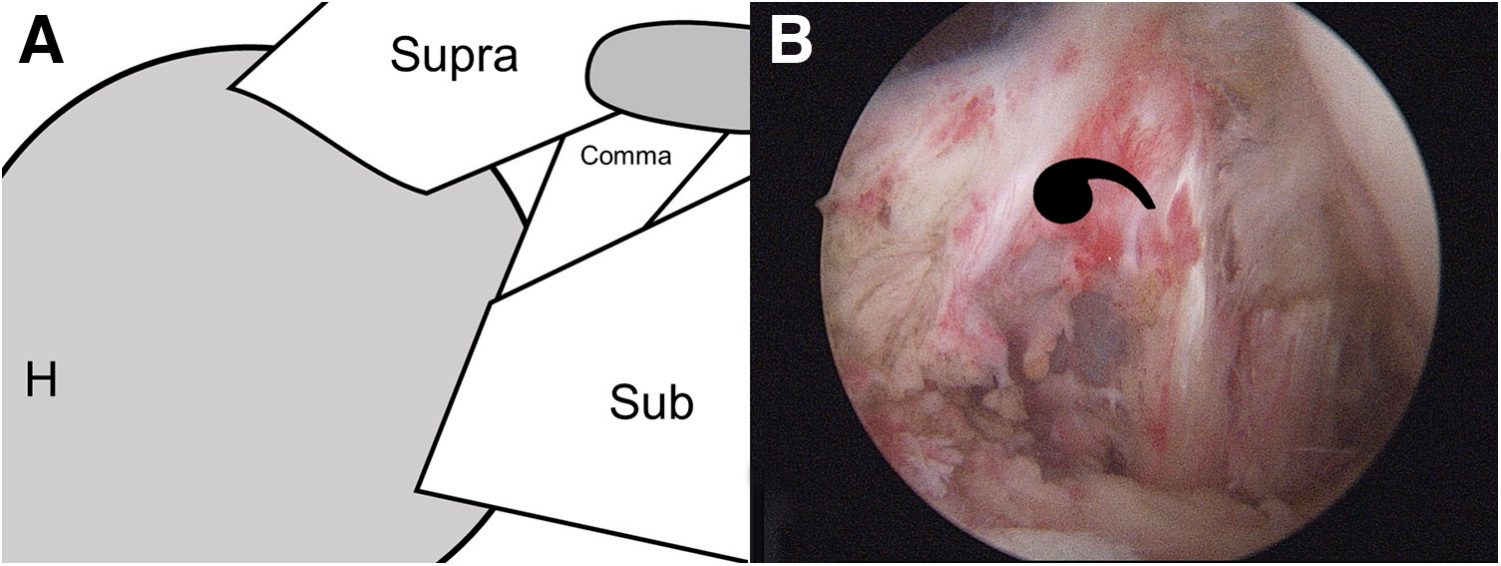
Illustration (A) and arthroscopic image (B) of the subscapularis tear in the right shoulder. The patient was placed in the lateral decubitus position. A standard posterior viewing portal and anterior portal were established. The retracted subscapularis and “comma sign,” known as the arc formed by a portion of the superior glenohumeral ligament/coracohumeral ligament complex, are noted. (H, humeral head; Sub, subscapularis; Supra, supraspinatus.)

To comprehensively visualize the subscapularis tendon and footprint, the humeral head is pulled posteriorly and 70° arthroscopy was is. Then, after subscapularis tear is confirmed (Fig 1), the footprint is prepared and debrided using a high-speed shaver (Stryker, Mahwah, NJ). To facilitate the examination and reduction of the subscapularis, a traction suture is applied using a spectrum suture hook (ConMed Linvatec, Largo, FL). Subsequently, after the stump is pulled laterally, the adhesive tissue in the subcoracoid region and part of the medial glenohumeral ligament are released.

**Fig 2:**
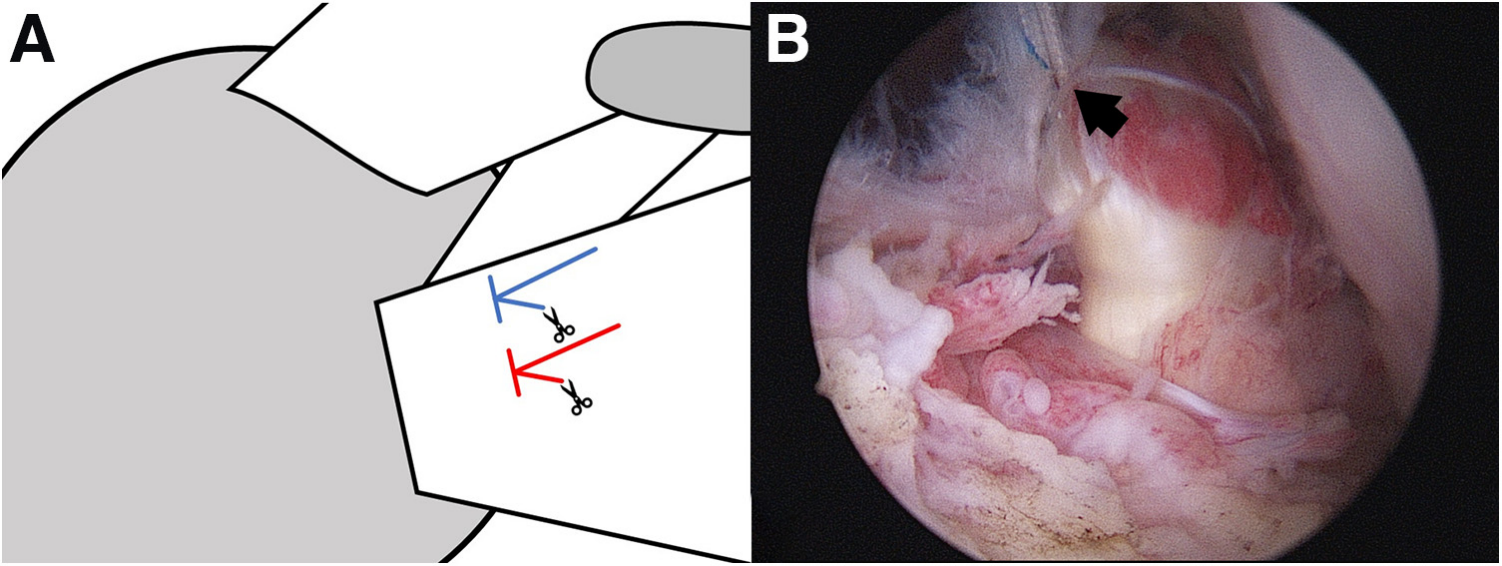
Illustration (A) and arthroscopic image (B) of the subscapularis repair. Two mattress sutures are used to repair the subscapularis tendon. one limb of each mattress suture is cut and the other is left. Thus, 2 suture limbs are retained (arrow in B).

One doubly loaded suture anchor (4.5 mm; Intai Technology, Taichung, Taiwan) is then applied in the lesser tuberosity. Next, a polydioxanone suture is passed though the tendon stump by a suture hook through the anterior portal.^4^ Thereafter, the braided suture is shuttled by polydioxanone, and all 4 braided sutures are sequentially passed through the tendon from the distal to the proximal position. Two mattress sutures are then achieved by tying both suture limbs. For further comma tissue repair, one limb of each mattress suture is cut and the other is left. Thus, 2 suture limbs are retained in the subacromial space (Fig 2).

### Comma Tissue Repair

**Fig 3:**
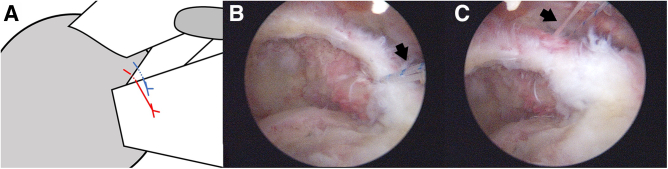
Illustration (A) and arthroscopic image before (B) and after (C) comma tissue repair. After subscapularis repair, 2 suture limbs are retained in the subacromial space. From lateral portal, the retained suture limbs (arrows in B and C) are then sequentially passed through the comma tissue using an antegrade suture passer.

After subscapularis repair, anterolateral and lateral portals are established by a spinal needle. Then, after the viewing scope is shifted to the lateral portal, bursectomy and limited acromioplasty are performed and the comma tissue is identified. The retained suture limbs are then sequentially passed through the comma tissue using an antegrade suture passer (Arthrex). Subsequently, after repair, the sutures are left in the cannula for further suture bridging (Fig 3).

### Supraspinatus Repair

**Fig 4:**
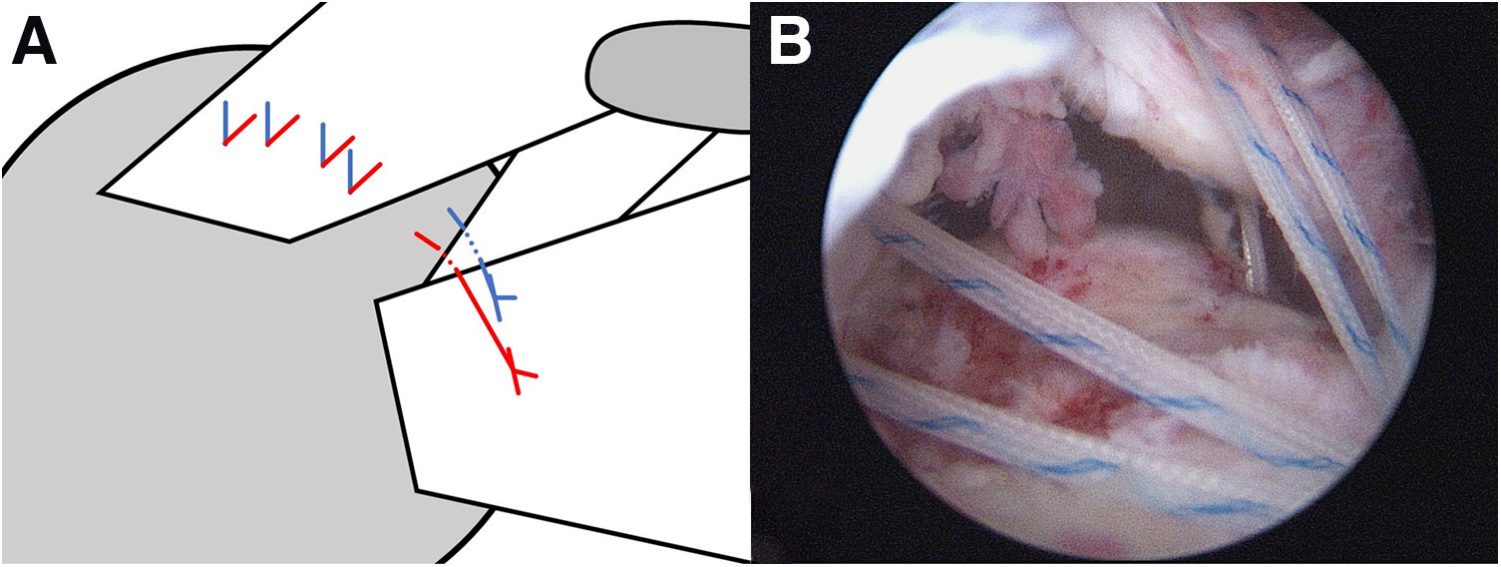
Illustration (A) and arthroscopic image (B) of supraspinatus repair. To repair the supraspinatus, a modified double-pulley suture bridge technique is adopted. Two doubly loaded suture anchors are placed at the edge of the cartilage and an ipsilateral pair of sutures are pass through the supraspinatus by a shuttle suture.

**Fig 5:**
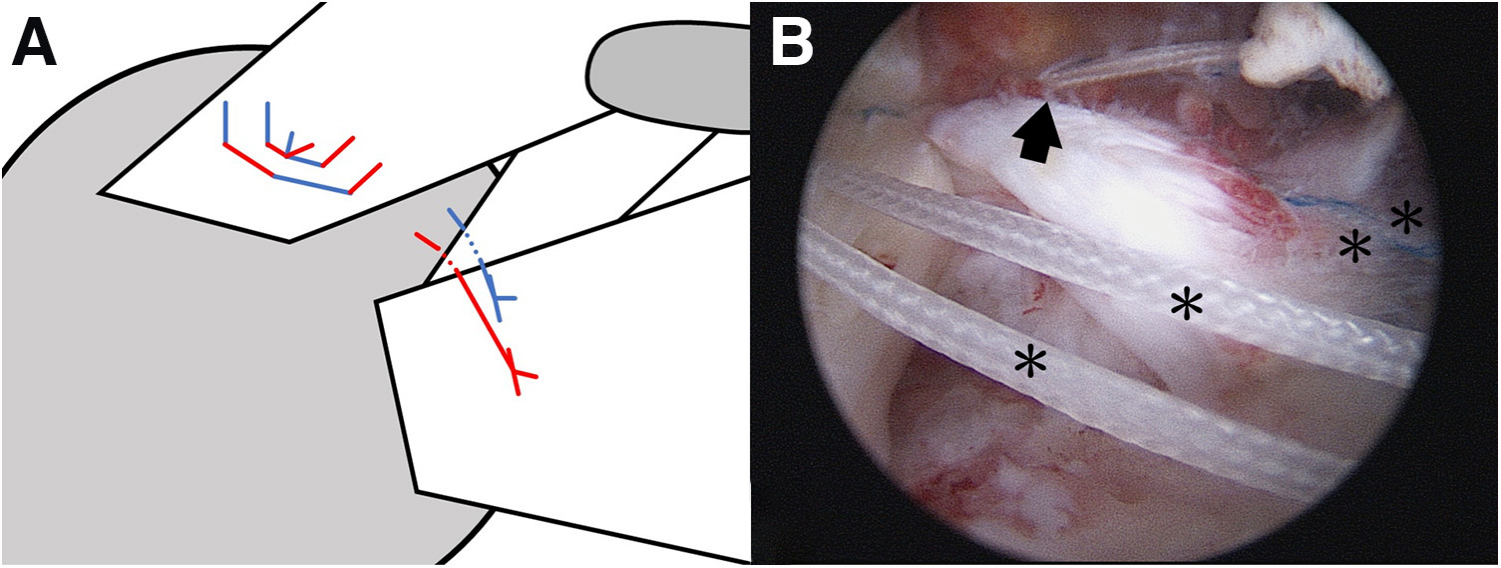
Illustration (A) and arthroscopic image (B) of the suture management of modified double pully supraspinatus repair. One suture limb from the anterior first pair and another limb from the fourth pair are pulled from the cannula. After the limbs are tied together, the ends of the sutures are cut. The remaining 2 limbs of the same suture from the second and third pairs are pulled. This allows the knot outside the cannula to be pulled inside and lie on the cuff. The 2 limbs are also tied (arrow in B). Meanwhile, the other suture limbs from the four pairs are retained without tying (∗ in B).

To repair the supraspinatus, a modified double-pulley suture bridge technique^5^ is adopted. First, 2 doubly loaded suture anchors are placed at the edge of the cartilage. Then, a shuttle suture is passed through the supraspinatus using an antegrade suture passer, and an ipsilateral pair of sutures are shuttled through the supraspinatus. This procedure is sequentially performed 4 times from the anterior to the posterior position (Fig 4). Thereafter, one suture limb from the anterior first pair and another limb from the fourth pair are pulled from the cannula. After the limbs are tied together, the ends of the sutures are cut. The remaining 2 limbs of the same suture from the second and third pairs are pulled. This allows the knot outside the cannula to be pulled inside and lie on the cuff. The 2 limbs are also tied. Meanwhile, the other suture limbs from the 4 pairs are retained without tying (Fig 5).

### Lateral Knotless Suture Anchor Placement

**Fig 6:**
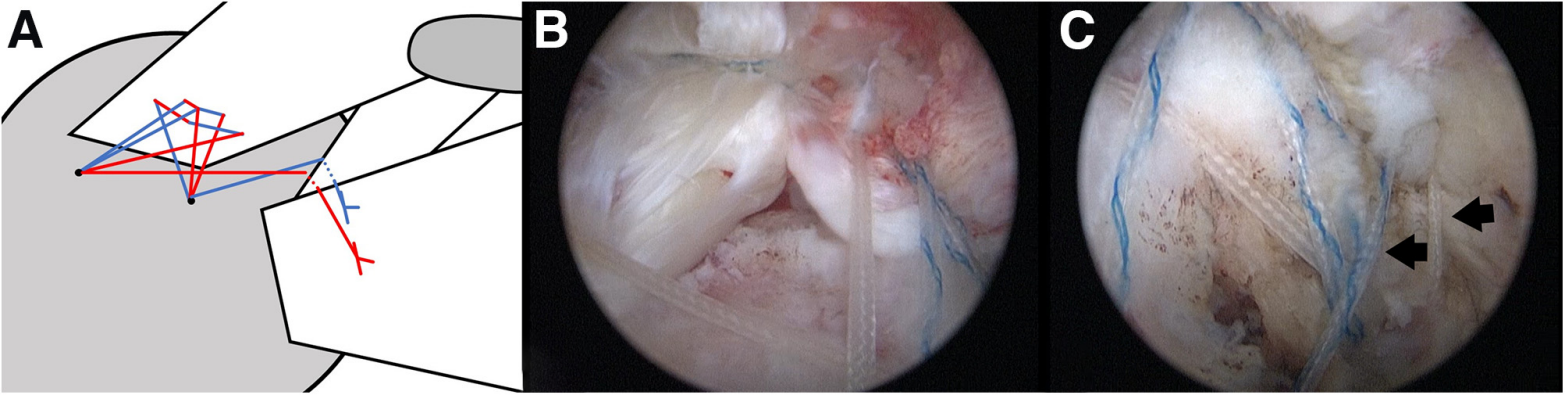
After suture management, 8 suture limbs remain: 2 from the subscapularis and comma tissue (arrow in C), 2 from the double-pulley suture, 2 directly from the anterior suture anchor, and 2 directly from the posterior suture anchor. One limb from each suture is then grabbed and pulled laterally down to the lateral aspect of the footprint to create a suture bridge using 2 knotless suture anchors.

After suture management, 8 suture limbs remain: 2 from the subscapularis and comma tissue, 2 from the double-pulley suture, 2 directly from the anterior suture anchor, and 2 directly from the posterior suture anchor. One limb from each suture is then grabbed and pulled laterally down to the lateral aspect of the footprint to create a suture bridge using two 4.5-mm PopLok anchors (ConMed Linvatec) (Fig 6).

## Discussion

Various surgical techniques are used for concomitant supraspinatus and subscapularis tear repair. For example, Bartl et al.^6^ repaired both the supraspinatus and subscapularis tendons using the single-row technique, whereas Lenart and Ticker^7^ suggested that subscapularis tendon repair with a single row is sufficient. In other studies, Chernchujit and Sharma^8^ and Greenspoon et al.^3^ repaired both tendons using the suture bridge technique. In the present study, we introduce merged lateral row modification of the suture bridge technique for simultaneous supraspinatus and subscapularis repair. We believe that this technique offers some advantages to previous methods (Table 1).

**Table 1:** Advantages, Limitations, and Risk of the Merged Lateral Row Modification of the Suture Bridge Technique for Simultaneous Supraspinatus and Subscapularis Repair

Advantages	Decreases the soft-tissue damage around the bicipital groove
	Reduces the number of suture anchors and is time-saving
	Concomitant comma tissue repair
	Adequate loaded suture in lateral row anchor
Limitations	A 70° arthroscopy is usually necessary
	Tenodesis or tenotomy is necessary
Risk	Risk of entangled sutures

First, our technique reduces the duration and cost of surgery and decreases soft-tissue damage around the bicipital groove. Compared with single-row repair, double-row repair requires the placement of more suture anchors for the lateral row in a restricted area of the greater tuberosity, which is challenging and time-consuming.^9^ With our technique, only 2 knotless suture anchors are necessary, which in turn decreases the operation time for suture management and anchor placement. In addition, our technique does not necessitate the placement of lateral row anchors in the bicipital groove and, therefore, helps preserve the soft tissue around the bicipital groove.

Second, our technique allows simultaneous repair of the comma tissue. The comma sign, which was first described by Lo and Burkhart,^10^ comprises parts of the coracohumeral ligament and SGHL.^11^ Some researchers have proposed that the stability of rotator cuff repair can be increased by preserving or even incorporating the comma sign into the repair.^10^^,^^12^ In their biomechanical study, Hackl et al.^13^ demonstrated that additional stabilization of the comma sign enhanced the primary stability of subscapularis tendon repair in anterosuperior rotator cuff tears in cadavers. With our technique, the comma tissue can be repaired without additional knot tying or suture anchor placement.

Third, the maximum number of loaded sutures differs depending on the design of the suture anchor, and the number of loaded sutures may affect the fixation strength.^14^ In their study, Corpus et al.^15^ reported a similar technique to fix the suture limbs of both tendons to lateral suture anchors. However, this technique involves 6 loaded sutures in one knotless suture anchor, which is not available in some types of knotless suture anchors. Cutting one suture limb in the subscapularis knot and using a double-pulley technique for supraspinatus repair allow obtaining only four suture limbs in each suture anchor, which is available in most types of suture anchors.

Overall, our technique has some limitations. First, 70° arthroscopy is usually required to identify the footprint of the subscapularis from the posterior viewing portal. Second, the suture limbs from the subscapularis knot span the bicipital groove. Thus, tenotomy or tenodesis should be performed on the long head of the biceps tendon. Third, repairing the subscapularis in the anterior single portal is technically challenging. Therefore, the order of pulling each suture limb should be well planned and suture tangling should be avoided.

## Conclusions

Merged lateral row modification of the suture bridge technique for simultaneous supraspinatus and subscapularis repair is an economic and time-preserving technique. Moreover, concomitant comma tissue repair increases the stability of and protects the repaired subscapularis. Hence, the procedure described herein is considered promising for the treatment of anterosuperior rotator cuff tear.

## Supplementary Data

ICMJE author disclosure forms

Video 1 In this video, we demonstrate the technique of a merged lateral row for suture bridge rotator cuff repair, in which the lateral suture of the supraspinatus and subscapularis is placed in the greater tuberosity. This technique can reduce both the duration and cost of surgery and decrease soft-tissue damage. It also can allow the “comma tissue” to be simultaneously repaired. This case involved the right shoulder of a 58-year-old man who had concomitant supraspinatus and subscapularis tear. The patient was placed in the lateral decubitus position with index arm traction by a 3-point traction system. A standard posterior viewing portal and anterior portal were established. Tenotomy of the long head of the biceps is usually performed. After subscapularis tear was confirmed, the footprint was prepared and debrided using a high-speed shaver. One doubly loaded suture anchor was then applied in the lesser tuberosity. Two mattress sutures were then achieved by tying both suture limbs. One limb of each mattress suture was cut and the other was left for further comma tissue repair. After subscapularis repair, anterolateral and lateral portals were established. The retained suture limbs were then sequentially passed through the comma tissue using antegrade suture passers. Subsequently, after repair, the sutures were left in the cannula for further suture bridging. To repair the supraspinatus, a modified double-pulley suture bridge technique was adopted. After suture management, 8 suture limbs remained: 2 from the subscapularis and comma tissue, 2 from the double-pulley suture, 2 directly from the anterior suture anchor, and 2 directly from the posterior suture anchor. One limb from each suture was then grabbed and pulled laterally down to the lateral aspect of the footprint to create a suture bridge using 2 knotless anchors.
